# Human Daily Breathing Monitoring via Analysis of CSI Ratio Trajectories for WiFi Link Pairs on the I/Q Plane

**DOI:** 10.3390/s24227352

**Published:** 2024-11-18

**Authors:** Wei Zhuang, Yuhang Lu, Yixian Shen, Jian Su

**Affiliations:** 1 School of Computer Science, Nanjing University of Information Science and Technology, Nanjing 210044, China; 202212490270@nuist.edu.cn (Y.L.); 202312490525@nuist.edu.cn (Y.S.); 2 School of Software, Nanjing University of Information Science and Technology, Nanjing 210044, China; sj890718@gmail.com

**Keywords:** breathing monitoring, WiFi sensing, channel state information, Internet of Things

## Abstract

The measurement of human breathing is crucial for assessing the condition of the body. It opens up possibilities for various intelligent applications, like advanced medical monitoring and sleep analysis. Conventional approaches relying on wearable devices tend to be expensive and inconvenient for users. Recent research has shown that inexpensive WiFi devices commonly available in the market can be utilized effectively for non-contact breathing monitoring. WiFi-based breathing monitoring is highly sensitive to motion during the breathing process. This sensitivity arises because current methods primarily rely on extracting breathing signals from the amplitude and phase variations of WiFi Channel State Information (CSI) during breathing. However, these variations can be masked by body movements, leading to inaccurate counting of breathing cycles. To address this issue, we propose a method for extracting breathing signals based on the trajectories of two-chain CSI ratios on the I/Q plane. This method accurately monitors breathing by tracking and identifying the inflection points of the CSI ratio samples’ trajectories on the I/Q plane throughout the breathing cycle. We propose a dispersion model to label and filter out CSI ratio samples representing significant motion interference, thereby enhancing the robustness of the breathing monitoring system. Furthermore, to obtain accurate breathing waveforms, we propose a method for fitting the trajectory curve of the CSI ratio samples. Based on the fitted curve, a breathing segment extraction algorithm is introduced, enabling precise breathing monitoring. Our experimental results demonstrate that this approach achieves minimal error and significantly enhances the accuracy of WiFi-based breathing monitoring.

## 1. Introduction

Breathing-related diseases, such as sleep apnea syndrome, have become a global health concern affecting over one billion people worldwide [1]. In developed countries, approximately 30 percent of individuals aged 70 and above suffer from respiratory system diseases [2]. Due to the intermittent nature of symptoms associated with respiratory system diseases, prolonged hospitalization for clinical observation and monitoring is often impractical. Therefore, accurate breathing monitoring is important in daily life environments such as homes and offices.

With the rapid advancement of IoT technology, the automation of various sectors including household, medical, and office settings has become a reality. The IoT facilitates real-time data collection and interaction by connecting various sensors and devices, thereby enabling precise monitoring and intelligent decision making. In the field of respiratory health, IoT technology provides a new avenue for home breathing monitoring. Deployment of sensor networks enables long-term, non-invasive monitoring of individual breathing status, thus providing robust support for the diagnosis and treatment of respiratory system diseases.

Nowadays, the methods used for breathing monitoring can be divided into contact breathing monitoring and non-contact breathing monitoring. Contact breathing monitoring technology typically employs sensors positioned in close proximity to the human body, which are less prone to environmental disturbances, thereby generally yielding more precise breathing signals compared to non-contact monitoring techniques. Contact breathing monitoring methods mostly use wearable embedded devices [3] or pressure sensors [4] to sense breathing. Long-term wear of wearable devices is not user-friendly for patients, who may experience discomfort and may move or even remove the device, thus making it challenging to obtain continuous breathing information. Using pressure sensors usually requires customizing the corresponding mattress, which is expensive to fabricate, and many patients consider it unnecessary. Therefore, although contact devices can obtain high-precision breathing information, they are invasive and often expensive, making them difficult to use in a daily environment.

Non-contact monitoring methods obviate the need for wearable sensors, offering a non-invasive and adaptable sensing approach suitable for long-term monitoring. Currently, the prevailing non-contact breathing monitoring methods include vision-based monitoring and wireless signal-based radio frequency monitoring. Vision-based methods use cameras to capture chest movements caused by human breathing, from video streams, to extract breathing signals [5,6]. However, these approaches depend on high lighting conditions and a Line Of Sight (LOS) environment, making them unsuitable for monitoring breathing status during sleep at night [7]. Additionally, vision-based methods raise privacy concerns [8]. Wireless signal-based sensing methods can typically perceive breathing from all directions, with lower risk of privacy breaches. For instance, researchers have proposed using Doppler radar, UWB radar [9], and FMCW radar [10] to sense breathing signals. These methods, similar to vision-based approaches, extract breathing signals by sensing chest movements through radar signals. Although radar-based breathing monitoring methods have demonstrated feasibility and have achieved high accuracy, their deployment costs are excessively high, making them difficult to implement in households and work environments. There is also a potential risk of radiation exposure.

WiFi signals, with their advantages of wide coverage, strong penetration, high reliability, and low cost, have been extensively studied by researchers and have achieved promising results in various sensing applications, such as gesture recognition, fall detection, crowd counting, and vital sign extraction. Early researchers typically used WiFi for breathing signal monitoring based on RSS (Received Signal Strength), capturing changes in signal strength caused by breathing. In reference [11], the UbiBreathe system was proposed to detect breathing pauses, using RSS. However, RSS is a coarse-grained wireless signal parameter that is highly susceptible to environmental interference, resulting in large measurement errors and making it unsuitable for monitoring subtle signals like breathing.

In contrast, CSI is a type of fine-grained information based on the physical layer, describing the Channel Frequency Response of multipath propagation and exhibiting good stability, making it well suited for breathing monitoring. In reference [12], Wu et al. proposed an innovative breathing detection method based on commercial WiFi equipment. However, this method primarily serves to distinguish between environments where only breathing movements are present and those where other human activities are also occurring. It does not encompass the recovery of breathing waveforms or the calculation of breathing rate. Zhang et al. [13] introduced the Fresnel zone model, elucidating its theoretical characteristics, thereby unveiling the propagation patterns of radio waves in WiFi and furnishing a theoretical framework for WiFi sensing. Zhang et al. [14] proposed the Wi-Cyclops system, which utilizes a single-antenna WiFi device to accurately capture subtle changes in CSI triggered by respiratory motion at room scale, thus enabling efficient respiratory detection at room scale. Burimas et al. [15], on the other hand, implemented WiFi-CSI-based respiration detection in a sleep environment, using an ESP32 single-board computer. This study highlighted the practicality of utilizing the widely available Wi-Fi CSI for non-invasive sleep monitoring, noting in particular the high value of this technique for the elderly and those suffering from sleep apnea symptoms. Dou et al. [16] utilized changes in Doppler spectral energy extracted from CSI collected by a Wi-Fi device to track chest displacement induced by respiratory movements. In turn, the consistency of the Doppler energy change period with the respiratory cycle was utilized to accurately obtain the respiratory rate. Lei et al. [17] proposed a respiration monitoring system that combines a commercial wireless router and a PC. By constructing the human chest cavity as a variable half-cylinder model, the periodic characteristics of the CSI respiration signals were deeply analyzed, thus realizing the accurate estimation of respiration frequency. The study also incorporated deep learning algorithms, to successfully achieve highly accurate classification of three breathing modes: normal breathing, apnea, and deep breathing.

In various studies [18,19,20,21], researchers have employed multiple antennas to monitor the breathing status of individuals during sleep. These studies have validated the complementary performance exhibited by antenna deployment strategies based on the Fresnel zone model in breathing monitoring. However, in literature [22], Wang et al. conducted an in-depth study, using the Fresnel zone model, and they explicitly pointed out the existence of blind spots in breathing monitoring. The presence of these blind spots may significantly reduce the accuracy of breathing monitoring, thus exerting a non-negligible adverse impact on the overall performance of breathing monitoring. Subsequently, Zeng et al. [23] discovered through their research that there exists a perfect complementarity between the amplitude and the phase of CSI. This signifies that in situations where amplitude sensing fails to function effectively, phase sensing can provide adequate compensation, and vice versa. In other words, their study suggests that when devising breathing monitoring solutions, the integration of both amplitude and phase information can effectively mitigate the impact of blind spots. However, in certain complex environments, multiple maxima and minima points of phase or amplitude may appear within a single cycle. This phenomenon results in an inability to accurately estimate the number of breathing cycles, even when combining amplitude and phase information. Consequently, the blind spot issue has not been completely resolved.

In this paper, to circumvent the blind spot issues that arise when solely relying on amplitude or phase information for breathing monitoring, we propose a method based on tracking CSI ratio trajectories on the I/Q plane. Our approach does not merely focus on amplitude, phase, or their combinations. Instead, we delve into the correspondence between the CSI ratio trajectories from different antenna pairs on the I/Q plane and the breathing process, aiming to achieve accurate breathing monitoring. The main contributions of this paper are as follows:

- We have found that although the trajectories of CSI ratios during breathing do not always exhibit regular arcs, they demonstrate a distinctive characteristic of significant angular turns alternating between exhalation and inhalation. Based on this feature, we propose a method to extract breathing signals using changes in CSI ratio trajectories on the I/Q plane.
- We propose a labeling technique based on a discrete model, which effectively filters out prominent motion interference. Additionally, we have devised a method for fitting the trajectory curves of CSI ratio sample points on the complex plane, accurately capturing curve inflection points that represent breathing transitions.
- We constructed a real-time breathing monitoring system, utilizing WiFi devices. We have validated that the system exhibits higher monitoring accuracy compared to the existing methods.

The remaining sections of this paper are organized as follows: In Section 2, the theoretical model of breathing monitoring based on WiFi-CSI is introduced. Section 3 presents our main work, including data preprocessing, the removal of interference from large movements, curve fitting, and the restoration of breathing waveforms. Our experimental results, including method validation and performance evaluation, are provided in Section 4. Finally, Section 5 draws the conclusion.

## 2. Theoretical Analysis

### 2.1. CSI Review

CSI estimation is one of the crucial steps in Orthogonal Frequency Division Multiplexing (OFDM) [24] signal demodulation, aimed at mitigating adverse effects such as frequency-selective fading in the actual channel. CSI falls under the domain of physical layer techniques and describes how signals propagate from the transmitter to the receiver in a communication link. It involves quantifying the attenuation factors along each transmission path to estimate channel characteristics, thereby providing more granular wireless channel information. CSI leverages OFDM to extract channel response information from the physical layer, delineating richer temporal and spectral structural information. Based on OFDM technology, CSI reduces multipath effects and enhances transmission efficiency.

In WiFi signals, fine-grained CSI based on OFDM describes the propagation of signals from the transmitter to the receiver at each subcarrier, showcasing combined effects such as scattering, fading, and power attenuation with distance. In the frequency domain, CSI can be represented as

(1) $$\vec{Y}=CSI\cdot \vec{X}+\vec{N}$$

where $\vec{Y}$ and $\vec{X}$ represent the received signal sequence and the transmitted signal sequence, respectively, and $\vec{N}$ represents additive white Gaussian noise. CSI denotes the channel matrix. To represent frequency response, each value in the CSI channel matrix is a complex number. In a single CSI record, there are T∗R∗N complex values, where *T* is the number of transmit antennas, *R* is the number of receive antennas, and *N* is the number of subcarriers. OFDM modulates the signal onto multiple parallel subcarriers for transmission. The CSI information obtained for each subcarrier at the receiver can be represented as $CSI=\left[\begin{array}{c} \mathrm{CSI}\;\left(f_{1}\right),\mathrm{CSI}\;\left(f_{2}\right),\ldots ,\mathrm{CSI}\;\left(f_{i}\right),\ldots ,\mathrm{CSI}\;\left(f_{N}\right) \end{array}\right]$, where $f_{i}$ represents the center frequency of the *i*th subcarrier.

In wireless channels, the scattering of signals in complex environments results in the simultaneous existence of multiple propagation paths. The transmitted signal experiences different time delays and signal attenuations on different paths, causing signal distortion and resulting in multipath effects. CSI information is highly susceptible to multipath effects during propagation; hence, the received signal is a superposition of signals from multiple paths. CSI identifies multipath signals through the Channel Impulse Response (CIR). In a non-time-varying system, the CIR can be represented by [25]

(2) $$h\left(\tau \right)=\sum_{i=1}^{N}a_{i}e^{-j\theta_{i}}\delta \left(\tau -\tau_{i}\right)+n\left(\tau \right)$$

where *N* is the total number of transmission paths, n(τ) is the Gaussian white noise present in the environment, $a_{i},\theta_{i},\tau_{i}$, respectively, represent the amplitude, phase, and delay of the ith path during signal transmission, and δ is the impulse response.

In general commercial devices, such as Intel 5300, it is not possible to accurately obtain the CIR. Assuming infinite bandwidth, the CIR and CFR are Fourier transform pairs. Therefore, the commonly adopted method is to model the channel using the Channel Frequency Response (CFR). The CFR is the ratio of the transmitted signal to the received signal: if we denote the frequency domain representations of the transmitted and received signals as $x(f_{k},t)$ and $y(f_{k},t)$, respectively, where $f_{k}$ is the carrier frequency, then the CFR $H(f_{k},t)$ measured at carrier frequency $f_{k}$ at time *t* can be expressed as

(3) $$H\left(f_{k},t\right)=\frac{y(f_{k},t)}{x(f_{k},t)}$$

$H(f_{k},t)$ contains both the amplitude and the phase information of the signal, namely,

(4) $$H\left(f_{k},t\right)=\left|H\left(f_{k}\right)\right|e^{j∠H(f_{k})}$$

where $\left|H(f_{k})\right|$ is the magnitude response of the *k*th channel at frequency, and where $∠H(f_{k})$ is the phase response.

### 2.2. Breathing Monitoring Model

**Figure 1:**
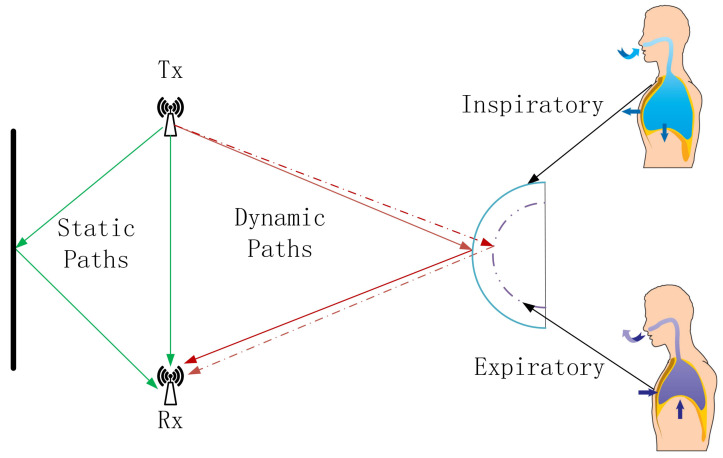
Breathing monitoring model.The green arrows indicate the LOS path signal and static reflection signal, while the red arrows indicate the dynamic reflection signal.

Human breathing is a continuous cyclic process consisting of exhalation and inhalation, and when the human body breathes, the thoracic cavity will form a continuous cyclic movement of expansion and contraction with exhalation and inhalation. We model the human thoracic cavity as a concentric cylinder during breathing, and the undulation of the thoracic cavity caused by human breathing is manifested as internal and external changes in the radius of the cylinder. Taking the human chest as the reflective surface, we consider the chest displacement caused by breathing, i.e., the change of the inner and outer radius of the modeled cylinder, as a moving object, which causes the change of the WiFi signal; then, we sense the breathing based on this change. Figure 1 shows the WiFi signal propagation path when there is a human body breathing in the current environment; at this time, the received signal is a composite signal containing the LOS path signal, the static reflection signal and the dynamic reflection signal. The received signal can be expressed as [26]

(5) $$H\left(f,t\right)=H_{s}\left(f\right)+H_{d}\left(f,t\right)$$

where $H_{s}$ represents the vector sum of the LOS path signal and the static reflection signal, and where $H_{d}$ is the vector sum of the dynamic object’s reflection signal, given by

(6) $$H_{d}\left(f,t\right)=A\left(f,t\right)e^{-j2\pi \frac{d(t)}{\lambda }}$$

where A(f,t) represents the attenuation of the dynamic component $H_{d}$, and where $-j2\pi \frac{d(t)}{\lambda }$ represents the signal propagation delay and d(t) represents the path length.

The dynamic path signal is the signal reflected from the WiFi signal through the human chest cavity, so the dynamic path signal path length will change periodically with the rise and fall of the chest cavity, which makes the amplitude and phase of the CSI in the received signal also change periodically, similar to the sinusoidal wave. The analysis of this correlation change can realize the use of WiFi to detect breathing, which provides new possibilities for non-invasive breathing monitoring. In the context of the Internet of Everything, this method is not only convenient and non-invasive, but also it can be seamlessly connected with other smart home devices to realize all-round home health monitoring.

## 3. Methods

**Figure 2:**
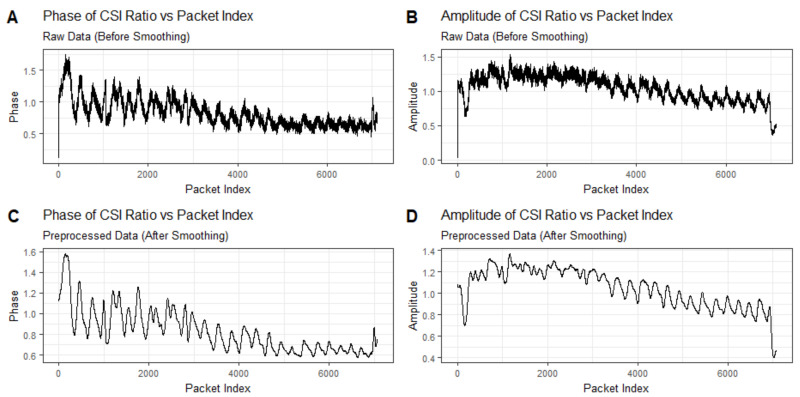
Time series plot of breathing signal detected using only amplitude or phase information. Subgraphs (**A**,**B**) exhibit the raw phase information and the original amplitude information of the CSI data, while subgraphs (**C**,**D**) depict smoothed versions of the phase and amplitude information.

Traditional CSI-based breathing monitoring methods typically rely on the amplitude or phase differences of the CSI to detect breathing patterns. As illustrated, Figure 2 comprises four subgraphs labeled A, B, C, and D. Subgraph A exhibits the raw phase information of the CSI data, while subgraph B presents the original amplitude information. These two subgraphs reveal the inherent complexity and variability of Wi-Fi channels, posing significant challenges for accurately detecting breathing patterns embedded within wireless signals. Subgraphs C and D depict smoothed versions of the phase and amplitude information, respectively, which can provide some indication of breathing, but which are still affected by irregular fluctuations that hinder effective monitoring.

In this paper, we introduce an innovative method for breathing monitoring, which is achieved by tracking the dynamic trajectories of CSI ratio sampling points on a complex plane.

**Figure 3:**
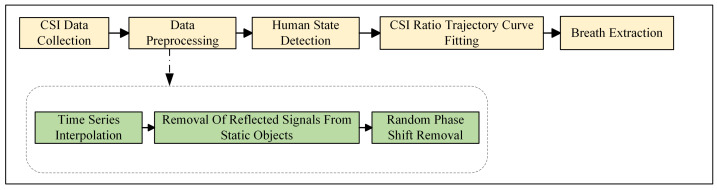
The flow diagram of our proposed method.

In Section 3.1, we describe how we initially preprocess the data, which includes time series interpolation and the removal of the signal components reflected by stationary objects. Subsequently, to address the random phase shift interference caused by the inherent hardware defects of WiFi devices, we effectively mitigate the impact of the random phase shifts by calculating the ratio of the CSI between two links, using this ratio as the observed signal for subsequent analysis. As described in Section 3.2, to reduce the interference caused by gross movements during the breathing process we propose a discretization model to identify significant signal changes caused by movements other than breathing. As detailed in Section 3.3, to accurately extract breathing waveforms we propose a curve fitting method capable of precisely fitting the trajectory curve of the CSI ratio sampling points on the complex plane. Utilizing the inflection points of the curve and the inherent characteristics of breathing, we introduce a breathing segment extraction algorithm that can accurately extract breathing waveforms. The flowchart of our proposed method is shown in Figure 3.

### 3.1. CSI Data Preprocessing

#### 3.1.1. Time Series Interpolation

Since the collected CSIs are not uniformly distributed in time, this means that the actual collection times of neighboring sampling points do not coincide. Therefore, in order to facilitate further analysis, it is necessary to transform the collected time series into a form with the same time interval. We propose a Gaussian regression algorithm called Continuous Time Field-Based to solve this problem. Assuming that each sampling point in the CSI is an observation from a normal distribution, Gaussian process regression [27] is used to model the time series data and process the irregularly spaced observations by interpolating the time series to create equally spaced observations.

In Gaussian process regression, the Radial Basis Function (RBF) kernel is chosen as the kernel function, which is defined as [28]

(7) $$k_{\mathrm{RBF}}\left(x,x^{\prime }\right)=\exp\left(-\frac{∥x-x^{\prime }{∥}^{2}}{2\ell^{2}}\right)$$

where *x* and $x^{\prime }$ are input data points and *ℓ* is the feature length scale.

The model first defines the covariance matrix of the observed data points through the kernel function, thus accurately capturing the correlation in the time series and quantifying its uncertainty. Next, we use great likelihood estimation to determine the hyperparameters in the kernel function to ensure that the model can better fit the actual data.

Subsequently, based on the identified kernel functions and input data points, we compute the covariance matrix. The covariance matrix, along with the observed data, is used to derive the posterior mean and covariance of the Gaussian process. These two parameters essentially form our interpolation function, where the a posteriori mean is used to interpolate the time series to generate observations with equal intervals; meanwhile, the a posteriori covariance provides an assessment of the uncertainty in these interpolated data and increases the confidence in the data. After establishing the Gaussian process regression model, we further apply the model to interpolate the raw time series. By predicting the expected value of the time series at equally spaced timestamps, we effectively fill in the gaps between irregularly spaced observations in the raw data and provide a continuous representation of the time series.

#### 3.1.2. Removal of Reflected Signals from Static Objects

As can be seen from Figure 1, when using WiFi signals for breathing monitoring, the signals received at the receiving end contain static path signals in addition to the dynamic path signals caused by breathing. The static path signal needs to be removed when performing breathing monitoring. The amplitude attenuation and phase offset of the WiFi signal propagated through the static path do not change with time and carrier frequency. Therefore, the components of the WiFi Channel State Information corresponding to this portion of the signal do not change in amplitude and phase with time at any carrier frequency $f_{k}$. That is, on the I/Q complex plane, for any carrier frequency $f_{k}$ the components of the WiFi Channel State Information corresponding to the static path signals are located at a fixed position with constant in-phase and quadrature components.

Since the static component does not vary with time, we estimate the static component on the subcarrier $f_{k}$ between the *i*th antenna and the *j*th antenna in the current scenario by collecting samples of the Channel State Information over time. Noting the starting moment as $t_{0}$ and the time window as *w*, the WiFi Channel State Information can be expressed as

(8) $$\begin{array}{cc} \sum_{t=t_{0}}^{t_{0}+W}H_{i,j}\left(f_{k},t\right) & =\sum_{t=t_{0}}^{t_{0}+W}H_{i,j}^{s}\left(f_{k},t\right)+H_{i,j}^{d}\left(f_{k},t\right)\\ \; & =\sum_{t=t_{0}}^{t_{0}+W}H_{i,j}^{s}\left(f_{k},t\right)+\sum_{t=t_{0}}^{t_{0}+W}H_{i,j}^{d}\left(f_{k},t\right) \end{array}$$

where $H_{i,j}^{s}\left(f_{k},t\right)$ is the static component and $H_{i,j}^{d}\left(f_{k},t\right)$ is the dynamic component. In the static environment, the dynamic component is 0; then,

(9) $$\sum_{t=t_{0}}^{t_{0}+W}H_{i,j}\left(f_{k},t\right)\approx \sum_{t=t_{0}}^{t_{0}+W}H_{i,j}^{s}\left(f_{k},t\right)+0$$

That is, the static component of the Channel State Information in the current environment may be estimated as the pre-collected Channel State Information within a window of time in the current environment, which may be obtained using smoothing from the Channel State Information collected at the receiving end.

#### 3.1.3. Random Phase Shift Removal

In practical wireless communication systems, different crystal oscillators are used at the transmitter and receiver ends, which leads to carrier frequency offset and packet detection delay in the Channel State Information, which can cause random fluctuations with large amplitude in the phase change caused by the motion of a dynamic object, and which greatly affects the breathing monitoring. To mitigate the issue of random phase errors, researchers have proposed hardware modifications, such as employing the same oscillator to provide clock signals for two distinct network cards to eliminate phase offsets. However, hardware-based modification methods pose challenges in their application to household WiFi devices, and the synchronization of clock signals between two wireless network cards also restricts the separation distance between the two WiFi communication devices [29].

Since different antennas on a receiving device share the same carrier frequency offset and packet detection delay, the dynamic path signal $H_{1,1}^{d}\left(f_{k},t\right)$ received by the first receiving antenna can be divided by that received by the second receiving antenna, $H_{1,2}^{d}\left(f_{k},t\right)$, to eliminate the carrier frequency offset and packet detection delay noise [30]. Where $H_{1,1}^{d}\left(f_{k},t\right)$ can be expressed as [31]

(10) $$H_{1,1}^{d}\left(f_{k},t\right)=a_{d,11}\left(f_{k},t\right)e^{-j2\pi \left(\int \Delta f_{c}\left(t\right)dt+f_{k}\tau_{d,11}\left(t\right)+\tau_{pdd}\left(t\right)f_{bb}\right)}$$

$\int \Delta f_{c}\left(t\right)dt$ and $\tau_{pdd}\left(t\right)f_{bb}$ denote the carrier frequency offset and packet detection delay, respectively:

(11) $$\begin{array}{c} \frac{H_{1,1}^{d}\left(f_{k},t\right)}{H_{1,2}^{d}\left(f_{k},t\right)}=\frac{a_{d,11}\left(f_{k},t\right)e^{-j2\pi \left(\int \Delta f_{c}\left(t\right)dt+f_{k}\tau_{d,11}\left(t\right)+\tau_{pdd}\left(t\right)f_{bb}\right)}}{a_{d,12}\left(f_{k},t\right)e^{-j2\pi \left(\int \Delta f_{c}\left(t\right)dt+f_{k}\tau_{d,12}\left(t\right)+\tau_{pdd}\left(t\right)f_{bb}\right)}}\\ =\frac{a_{d,11}\left(f_{k},t\right)}{a_{d,12}\left(f_{k},t\right)}e^{-j2\pi \left(f_{k}\tau_{d,11}\left(t\right)-f_{k}\tau_{d,12}\left(t\right)\right)} \end{array}$$

It can be shown that the carrier frequency offset and packet detection delay noise are not included in the ratio of the Channel State Information corresponding to the signals reflected by the dynamic object from the same transmitting antenna $Tx_{1}$ to the receiving antennas $Rx_{1}$ and $Rx_{2}$. This ratio is a complex number with modulus $\frac{a_{d,11}\left(f_{k},t\right)}{a_{d,12}\left(f_{k},t\right)}$ and phase $2\pi \left(f_{k}\tau_{d,11}\left(t\right)-f_{k}\tau_{d,12}\left(t\right)\right)$. We utilize the trajectory of this ratio in the complex plane for breathing monitoring in subsequent analyses.

### 3.2. Human State Detection Algorithm

In Section 3.1, we demonstrated how we can successfully eliminate the interference caused by random phase shifts by calculating the ratio of the CSI values between different links, which improves the stability of the signal. Next, we demonstrate how we utilize this ratio to effectively detect breathing signals.

**Figure 4:**
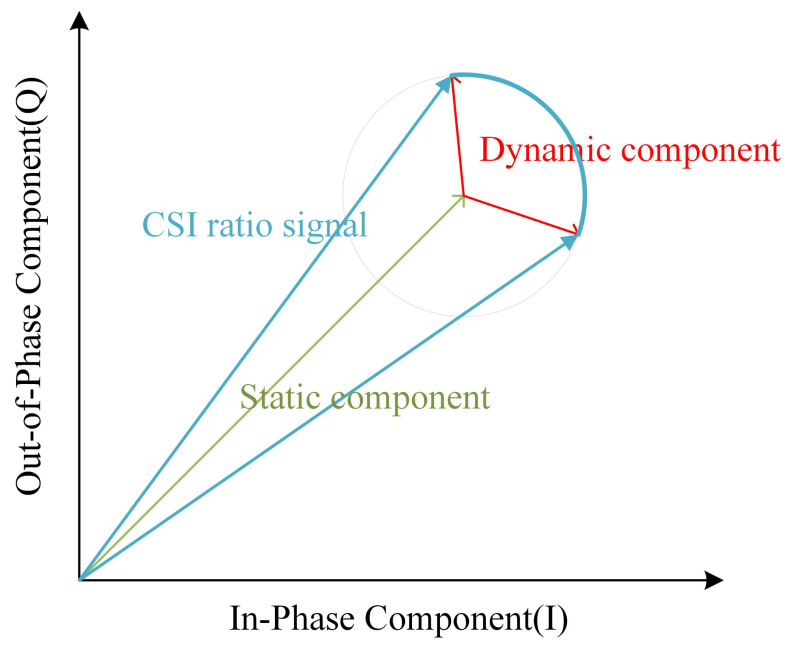
Vector representation diagram of CSI ratio signal.

**Figure 5:**
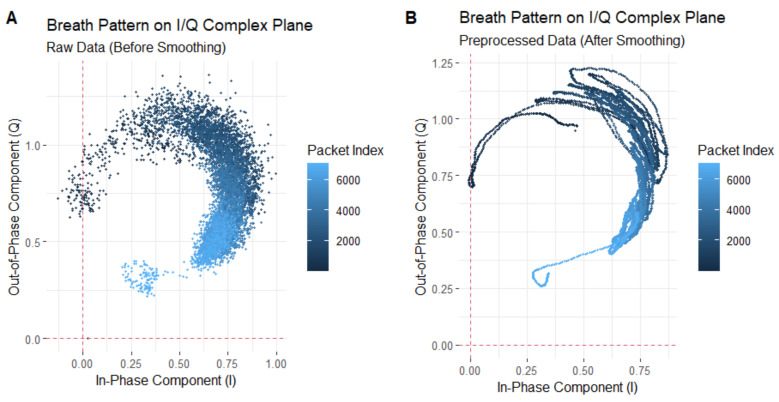
Breathing pattern on I/Q complex plane: (**A**) represents the unsmoothed trajectory, while (**B**) represents the smoothed trajectory.

Figure 4 is a graph displaying a vector representation of the CSI ratio signal. In the figure, the green line depicts the static component of the CSI ratio, the red line demonstrates the dynamic component, and the blue line represents the overall ratio signal. Due to the periodic movement of the chest cavity caused by human breathing, the dynamic path undergoes periodic changes. This variation leads to the evolution of the overall CSI ratio signal in the form of a unit circle on the complex plane. In other words, in ideal conditions the trajectory of the ratio signal appears as an arc, just as depicted by the blue arc portion in Figure 4:

However, upon closer observation of the trajectories of the CSI ratios on the complex plane during breathing, we find that these trajectories do not always form regular arcs. Figure 5 illustrates the breathing patterns on the I/Q complex plane before and after smoothing. In Panel A, we see a segment of 60-s CSI ratio sample data. In this figure, the horizontal axis represents the in-phase component values, while the vertical axis represents the quadrature component values. Each point is color-coded according to the temporal order of the sample points. Panel B reveals the layout of the CSI data points after smoothing filtering, elucidating the variation patterns of the CSI ratio sample points in the in-phase and quadrature planes. Although these trajectories do not always form standard arcs, but rather exhibit a spiral pattern within the breathing cycle, we note significant large-angle turning points during the transition from exhalation to inhalation in these spiral trajectories. These turning points accurately reflect the number of breathing cycles. Based on this discovery, we can achieve breathing monitoring by analyzing the trajectories of the CSI ratios.

However, when there is substantial movement interference in the environment, the distribution of points on the complex plane becomes chaotic and disorganized, which seriously affects the accurate recognition of the breathing signals.

In order to address this issue, we conduct a data cleaning effort, targeting sample points affected by significant motion interference based on the trajectory characteristics of the CSI ratio sample points on the complex plane. Through precise labeling and filtration of the CSI sample points that represent significant movement interference, we enhance the data purity effectively, laying a reliable foundation for subsequent accurate recovery of breathing signal analysis. The specific methods are outlined as follows: We propose a dissimilarity formula for labeling and filtering out CSI ratio sample points associated with significant motion interference. For a set of CSI data obtained through Rx transmitting antennas and Tx receiving antennas, comprising Rx×Tx×N subcarriers, each pair of transmitting and receiving antennas forms a link. $Q_{ij}^{kl}$ represents a sample point of the CSI link ratio after removing random phase offsets. The trajectory of the sample points over a period of time is depicted on the complex plane, with a time window *W* set to calculate the dispersion of these points. The following formula represents the dispersion, where Rx denotes the number of transmitting antennas, Tx represents the number of receiving antennas, $N_{f}$ is the number of subcarriers at the center frequency *f*, *W* is the size of the time window, and *m* denotes the *m*th subcarrier:

(12) $$\begin{array}{cc} D & =\sum_{i,j\in Rx}\sum_{k,l\in Tx}I\left((i\neq k)\vee (i\neq l)\vee (j\neq k)\vee (j\neq l)\right)\frac{1}{\left|N_{f}\right|}\sum_{m\in N_{f}}\frac{1}{W-1}\sum_{r=1}^{W}\\ & {\left[Q_{ij}^{kl}\left(f_{m},t_{n+r}\right)-\bar{Q_{ij}^{kl}\left(f_{m},t_{n}\right)}\right]}^{2} \end{array}$$

where $I\left((i\neq k)\vee (i\neq l)\vee (j\neq k)\vee (j\neq l)\right)$ is an indicator function, which yields a value of 1 if the conditions inside are met; otherwise, it is 0; k,l,i,j, respectively, represent the *k*th and *i*th transmitting antennas, and the lst and *j*th receiving antennas. This indicator function ensures that the two links are different.

(13) $$Q_{ij}^{kl}\left(f_{m},t_{n+r}\right)=\frac{H_{kl}\left(f_{m},t_{n+r}\right)}{H_{ij}\left(f_{m},t_{n+r}\right)}$$

The above expression represents the CSI ratio between two different links. $H_{ij}\left(f_{m},t_{n+r}\right)$ denotes the CSI (a complex number) from the *i*th transmitting antenna to the *j*th receiving antenna at time n+r on the subcarrier frequency $f_{m}$, where *n* is a positive integer, $t_{n}$ represents the starting time of the current time window, and $t_{\left(n+r\right)}$ represents the *r*th moment starting from the starting time *n* of the current time window.

(14) $$\bar{Q_{ij}^{kl}\left(f_{m},t_{n}\right)}=\frac{1}{w}\sum_{r=1}^{w}Q_{ij}^{kl}\left(f_{m},t_{n+r}\right)$$

The above expression represents the mean of the sample points within the current time window.

**Figure 6:**
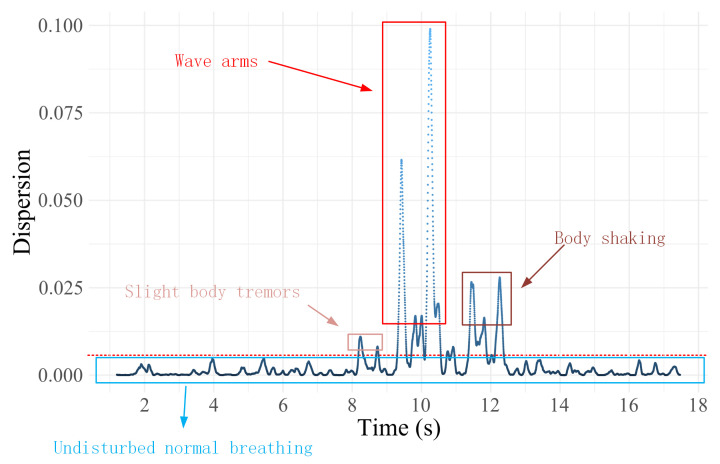
Dispersion of sample points of CSI ratio trajectories for breathing processes containing large action perturbations.

As illustrated in Figure 6, the plot depicts the variation in dispersion of the CSI ratio sample points during an 18-s breathing process for the participant. Throughout this period, the participant exhibits significant movements, including slight body tremors, arm waving, and body swaying. Specifically, between the 8th and 9th s, when the participant experiences slight body tremors, the dispersion of the sample points is slightly elevated compared to normal breathing, albeit not significantly. This is due to the similarity in magnitude between the slight body tremors and the breathing-induced chest movements. However, from the 9th to the 11th s, during arm waving, there is a substantial increase in sample point dispersion, far exceeding that observed during normal, undisturbed breathing. This indicates that the large arm movements overshadow the breathing-induced chest movements. Subsequently, from the 11th to the 13th s, as the participant ceases arm waving and exhibits minimal body swaying, the dispersion decreases but remains significantly higher than that of normal breathing.

Based on these observations and analyses, we employ the dispersion value observed during normal breathing as a reference, and we set a threshold at 0.001, to identify and label sample points exceeding this range as interference points. These points primarily represent movements unrelated to breathing-induced chest movements. The implementation process of this method is that for the preprocessed CSI ratio sample points, we first calculate the degree of dispersion of these sample points. Subsequently, based on a preset threshold criterion, a determination is made as to whether the current sample point is a discrete point. If the judgment result is a non-discrete point, the iteration continues to the next sample point for detection; on the contrary, if it is confirmed to be a discrete point, the point will be explicitly labeled and excluded from the subsequent trajectory curve fitting and respiratory signal extraction process. This approach enables more precise identification and differentiation of significant motion interferences during breathing, providing a solid foundation for accurate analysis of breathing waveforms in subsequent studies.

### 3.3. CSI Ratio Trajectory Curve Fitting and Breathing Extraction Algorithm

After the effective filtering of large-motion interference noise in the previous step, we successfully obtain the complex plane trajectories that can clearly characterize the breathing pattern. According to the inherent characteristics of human breathing, when the human body transitions between exhalation and inhalation, as well as between inhalation and exhalation during breathing, a short pause phenomenon occurs in the chest cavity. Of particular note, the pause for the transition between expiration and inspiration is usually slightly longer than the pause for the transition between inhalation and exhalation. This subtle breathing characteristic is accurately represented in the trajectories of the CSI ratio sampling points on the complex plane.

**Figure 7:**
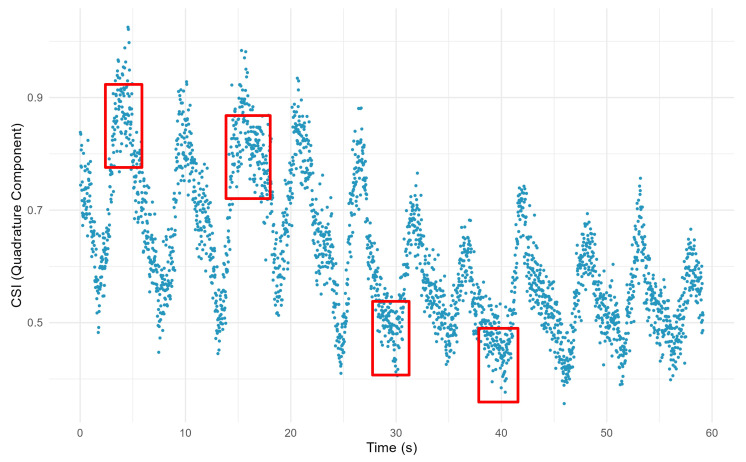
The trajectory of CSI ratio sample points containing breathing information. The red boxes mark areas where some of the CSI ratio sample points are clustered and where the breath state is altered.

Specifically, during breathing transitions, we observe a large number of sampling points clustered on the complex plane at specific ranges. The distribution pattern of these aggregated points closely corresponds to the peaks and troughs in the breathing waveform, providing us with an intuitive and accurate characterization of the breathing signal. To further reveal this dynamic process, we introduce the time axis as a reference. Under the detailed observation in the time dimension, the above breathing characteristics and their performance on the complex plane trajectory are clearly shown, as shown in Figure 7:

The areas where the CSI ratio sample points are clustered, as indicated by the red boxes in the figure, are key to identifying the breathing transition process. The denser aggregation of points in the trough region mainly reflects the transition phase of expiratory-to-inspiratory transition, while the sparser aggregation of points in the peak region corresponds to the process of inspiratory-to-expiratory transition. The precise identification of these aggregated points is crucial for the accuracy and reliability of breathing monitoring.

However, directly relying on these discrete sample points to recognize the inflection point information of breathing transitions is a challenging task. To overcome this difficulty, we propose an effective curve fitting method. By curve fitting the motion trajectories of these CSI ratio sample points on the complex plane, we are able to identify the inflection points of the trajectory curves more clearly and accurately, thus realizing the high-precision monitoring of breathing states.

For any $j,l\in R_{x},\;i\in T_{x},\;f_{k}\in N_{f}$, we determine the optimal coefficient vector β that minimizes the error, using the following formula:

(15) $$\arg\min_{\beta }\sum_{r=1}^{w}{\left[\sum_{d=1}^{p}\beta_{r}\phi_{d}\left(t_{n+r}\right)-Q_{il}^{ij}\left(f_{k},t_{n+r}\right)\right]}^{2}$$

where $\sum_{d=1}^{p}\beta_{r}\phi_{d}\left(t_{n+r}\right)$ represents the fitted curve;

(16) $$\phi_{d}\left(t_{n+r}\right)=\exp\left(-\frac{{\left(t_{n+r}-\frac{d}{p+1}\right)}^{2}}{2s^{2}}\right)$$

where *p* and *s* are hyperparameters; *p* denotes the order of the kernel function, while *s* controls the smoothness of the fitted curve.

**Figure 8:**
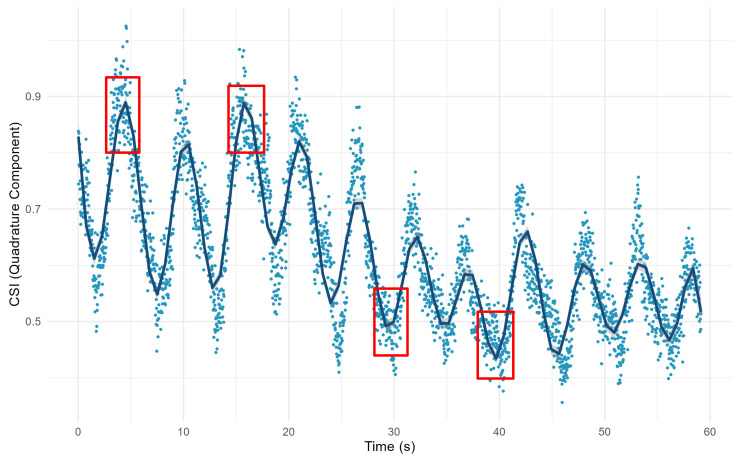
Trajectory of CSI ratio after curve fitting. The red boxes indicate part of the breath transition region after fitting the curve.

As illustrated in Figure 8, the trajectory of the CSI ratio is presented after an effective curve fitting process. Through this fitting process, we are able to identify the inflection points in the trajectory more accurately. After that, we propose a respiratory segment extraction algorithm, which is used to effectively reconstruct the respiratory waveform for accurate breathing monitoring.

The breathing segment extraction algorithm is shown in Algorithm 1. Remember that the trajectory distance of the Channel State Information ratio $Q_{il}^{ij}\left(f_{k},t\right)$ on the subcarrier $f_{k}$ moving on the complex plane from the moment $t_{0}$ to the moment $t_{0}+\Delta t$ is $\Delta L=\int_{t=t_{0}}^{t=t_{0}+\Delta t}\left|\frac{d}{dt}\left(Q_{il}^{ij}\left(f_{k},t\right)\right)\right|ds$. We assume that the set of transition phase moments identified by the algorithm is $T=\{t_{0}+\Delta t_{1},t_{0}+\Delta t_{2},\ldots ,t_{0}+\Delta t_{m},\ldots ,t_{0}+\Delta t_{n}\}$, where $\Delta t_{m}$ is an integer multiple of Δt and is the minimum time interval considered by the algorithms in this section, and according to Algorithm 1 the signal $S_{il}^{ij}\left(f_{k},t\right)\in R^{n}$ characterizing respiration is obtained from the sequence $Q_{il}^{ij}\left(f_{k},t\right)$.
**Algorithm 1** Breathing Segment Extraction Algorithm**Input:** 
$Q=\left[\begin{array}{c} Q_{i,l}^{i,j}\left(f_{k},t_{0}\right),\ldots ,Q_{i,l}^{i,j}\left(f_{k},t_{0}+\Delta t\right),\ldots ,Q_{i,l}^{i,j}\left(f_{k},t_{0}+n\times \Delta t\right) \end{array}\right]$
**Output:**  
$S=\left[S_{i,l}^{i,j}\left(f_{k},t_{0}+\Delta t\right),S_{i,l}^{i,j}\left(f_{k},t_{0}+2\times \Delta t\right),\ldots ,S_{i,l}^{i,j}\left(f_{k},t_{0}+n\times \Delta t\right)\right]$
  1:Initialize an all-zero sequence *S* of length *n* with initialization flag=1.  2:Step 1: Fit *Q*, using a spline curve to obtain a smoothed CSI ratio sequence $\hat{Q}$.  3:Step 2:  4:**for** i=2 **to** *n* **do**  5:     $\Delta \hat{Q}={\hat{Q}}_{il}^{ij}\left(f_{k},t_{0}+i\times \Delta t\right)-{\hat{Q}}_{il}^{ij}\left(f_{k},t_{0}+\left(i-1\right)\times \Delta t\right)$  6:     θ=arctan(Re(ΔQ)Im(ΔQ^))  7:     **if** $\theta >\frac{2}{3}\pi$ **then**  8:       $T=T\cup \{t_{0}+i\times \Delta t\}$  9:     **end if**  10:**end for**  11:Step 3:  12:**for** i=2 **to** *n* **do**  13:     **if** $(t_{0}+i\times \Delta t)\in T$ **then**  14:       flag=−flag  15:     **end if**  16:     Calculate the length of the smoothed CSI ratio moving trajectory on the complex plane from moment $t_{0}+\left(i-1\right)\times \Delta t$ to moment $t_{0}+i\times \Delta t$: $\Delta L=\int_{t=t_{0}+\left(i-1\right)\times \Delta t}^{t=t_{0}+i\times \Delta t}\left|\frac{d}{dt}\left({\hat{Q}}_{il}^{ij}\left(f_{k},t\right)\right)\right|ds$  17:     Update *S*: S[i]=S[i−1]+flag×ΔL  18:**end** **for**  19:**return** *S*


## 4. Experiment and Evaluation

### 4.1. Data Collection

**Figure 9:**
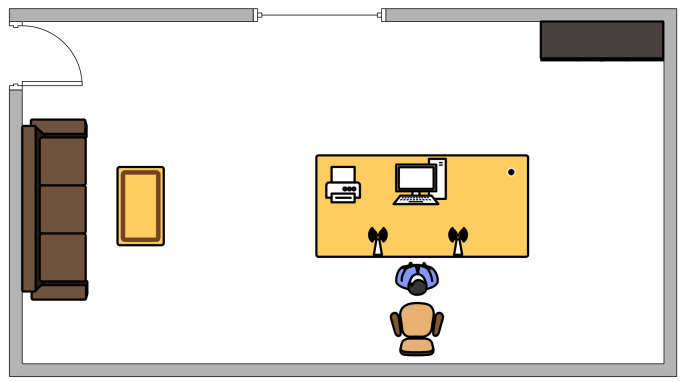
Schematic diagram of the scene.

We conducted extensive experiments, to evaluate the performance of our proposed method and compare it with existing methods. The experimental setup was as follows:

(1) Data collection: To evaluate the performance of our proposed method for monitoring breathing, we used an HKH-11C+ wearable respiratory wave sensor made of piezoelectric material for acquiring the real respiratory signals of the human body. The experimental environment of this study was set up in a typical office scenario, in which the transmitter and receiver ends of the router were deployed at locations on both sides of the desk, while the subjects participating in the experiment wore wearable respiratory sensors, sitting on the side of the router’s horizontal connection line, to be monitored. The exact layout is shown in Figure 9. We collected respiratory signals from four individuals for 120 s each. Afterwards, the performance was evaluated by comparing the respiratory signals extracted using the four different methods with the monitoring results of the piezoresistive respiratory belts worn by the four individuals.

(2) Hardware setup: In this experiment, we utilized two off-the-shelf WiFi routers. The hardware platform included a TP-LINK WDR4310 wireless router for collecting wireless Channel State Information, a server for data processing and detection, and a client device for displaying recognition results. The software platform consisted of the OpenWRT operating system for the wireless router, CentOS 7 for the detection and recognition server, and Windows 10 for the client device. To facilitate the collection of the Channel State Information, the original firmware on the wireless router was replaced with a modified OpenWRT system firmware containing customized drivers. The program running on the wireless router was coded in C language. Due to the limited computational and storage resources of the router itself, the program was cross-compiled on a server running Ubuntu 16.04. The CSI acquisition system hardware parameters are shown in Table 1:

### 4.2. Method Validation

In Section 3, we elaborated on an innovative breathing monitoring method that achieves breathing monitoring by tracking the trajectory of CSI ratio sample points on the complex plane. For this section, we conducted an in-depth analysis of the evident limitations present when relying solely on the amplitude or phase of a single CSI ratio sample point for breathing monitoring. Furthermore, we validated the effectiveness of our proposed approach through experimentation.

**Figure 10:**
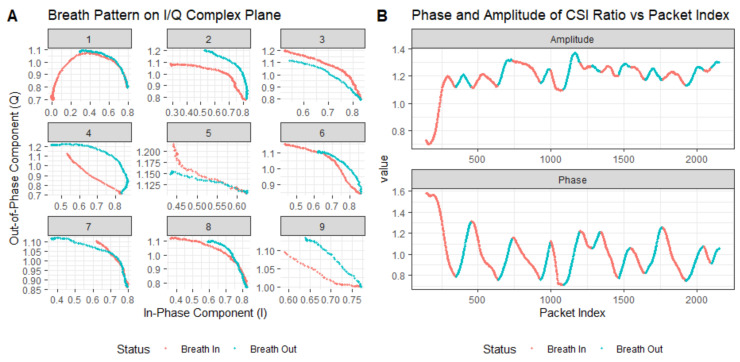
Extracted breathing pattern and recovered breathing signal using trajectory tracking method: (**A**) denotes the respiratory pattern on the I/Q complex plane and (**B**) represents the curves of the CSI ratio and phase changes during breathing.

Figure 5 shows the trajectory of the CSI ratio sampling points on the complex plane corresponding to the 60-s breathing process. From this trajectory, we extracted the ratio trajectories corresponding to nine consecutive breathing cycles, for analysis. In Figure 10, Figure A depicts the trajectory of the CSI ratio on the I/Q plane corresponding to the “inhale” and “exhale” processes of nine breathing cycles. An evident inflection point is observable in the CSI ratio on the I/Q plane during both the “inhale” and “exhale” processes. Moving to Figure B’s upper graph, it displays the amplitude variation curve of the CSI ratio over time for the nine breathing cycles. Despite there being only nine breathing cycles in this timeframe, the curve exhibited 12 local maximum points. This suggests that accurately determining the number of breaths solely based on the amplitude variation of the CSI ratio can be challenging. During a breathing cycle, the amplitude of the CSI ratio may not consistently vary, resulting in two or even three local maximum points within a single cycle.

The lower graph in Figure B illustrates the phase variation curve of the CSI ratio over time for the nine breathing cycles. Notably, it demonstrates a decrease in the phase during “inhale” and an increase during “exhale”. While this indicates that within this timeframe, the phase based on the CSI ratio could accurately estimate the breathing cycle, this was based on the premise of no bodily motion interference. However, the presence of bodily motion interference can lead to phase distortion in the CSI ratio, consequently affecting the accurate estimation of the breathing cycle.

**Figure 11:**
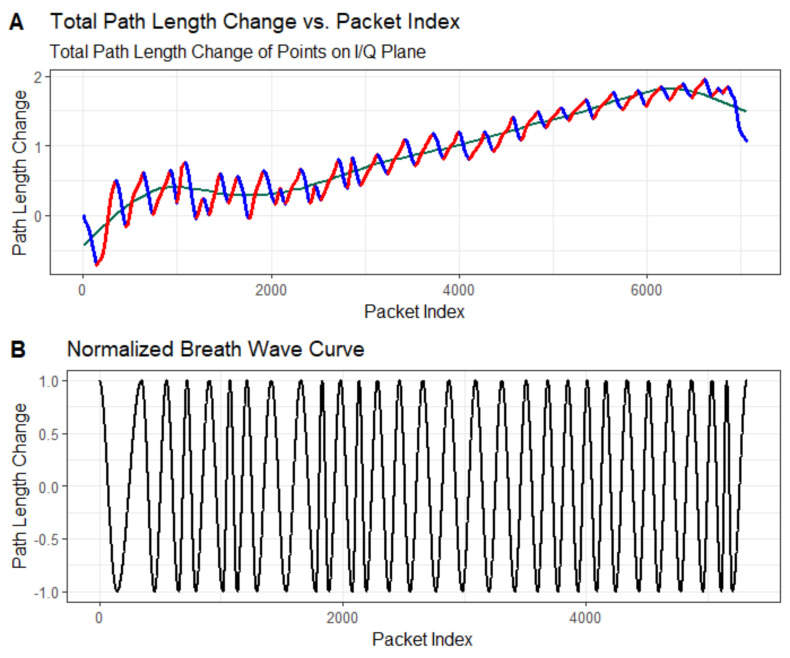
Path length change of extracted breathing wave and the recovered breathing signal: (**A**) denotes the total path length of the change at each point on the I/Q plane, and (**B**) indicates the normalized breathing waveform.

According to the method proposed in Section 3, we used the inflection points obtained from curve fitting to track the motion trajectories of the ratio sampling points and, thus, to monitor breathing. In Figure 11, we show the trajectory of the CSI ratio sampling point on the I/Q plane, and the corresponding curve is shown in Panel A. Subsequently, we normalized the data, and we display the respiration waveforms in Panel B, which clearly shows the frequency of change of the breathing waveforms over a specific time period.

### 4.3. Performance Evaluation

For this section, we evaluated the monitoring accuracy of our proposed method and compare it with existing methods, including Wi-Breath [32], WiCare [33], and PresSense [34]. Our evaluation included two different scenarios: firstly, the human chest orientation remains the same and the position changes; secondly, the human chest position remains the same and the orientation changes.

#### 4.3.1. Evaluation Indicators

In order to compare the accuracy between the breathing signals generated by a WiFi-based device and the signals collected by a wearable breathing monitoring device, for this paper we used three metrics—Average Accuracy (ACC), Mean Absolute Error (MAE), and Absolute Error Rate (AER)—to denote the difference between the two.

The Average Accuracy in a single breathing test was calculated as follows:

(17) $$ACC=AVR(\frac{t_{wearable}-\left|t_{wearable}-t_{CSI}\right|}{t_{wearable}})$$

where $t_{wearable}$ was the respiratory interval from the wearable breathing detection device, $t_{CSI}$ was the respiratory interval detected by the WiFi device, and AVR was the average value over one minute.

The MAE was calculated as follows:

(18) $$MAE=\frac{\sum_{t=1}^{N}\left|\varphi_{t}-g_{t}\right|}{N}$$

where $\varphi_{t}$ was the normalized respiratory signal extracted from the WiFi device, $g_{t}$ was the normalized respiratory signal collected by the wearable breathing monitoring device, and *N* was the length of the breathing sequence. This metric could reflect the proximity of the breathing signals extracted from the WiFi-based breathing monitoring system and the wearable-based breathing monitoring device.

The AER was calculated as follows:

(19) $$AER=|R_{wearable}-R_{CSI}|$$

where $R_{wearable}$ denoted the frequency of the breathing signals (breaths per minute) extracted from the wearable breathing-monitoring devices, and where $R_{CSI}$ denoted the frequency of the breathing signals extracted from the WiFi devices. This metric could be used to assess the accuracy of WiFi-based breathing frequency monitoring.

#### 4.3.2. Assessment of Different Orientations of the Human Chest

**Figure 12:**
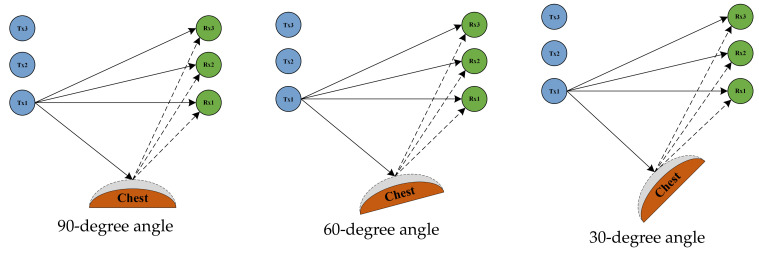
The human chest at an angle of 90 degrees, 60 degrees, and 30 degrees to the vertical plane.

**Figure 13:**
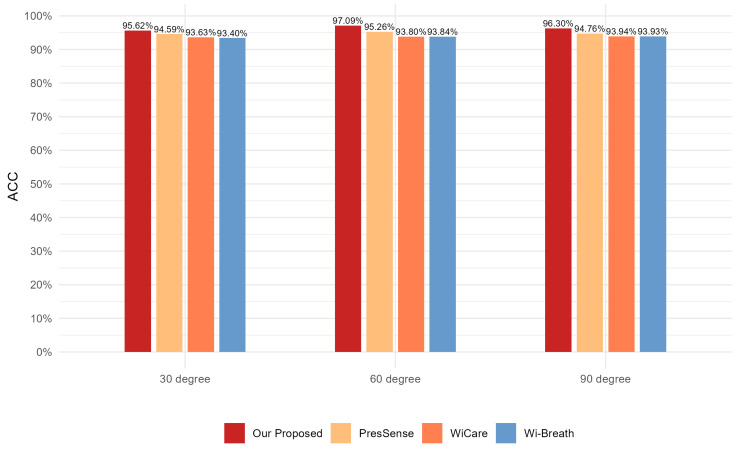
Comparison of ACC for four breathing monitoring methods in three different orientations.

**Figure 14:**
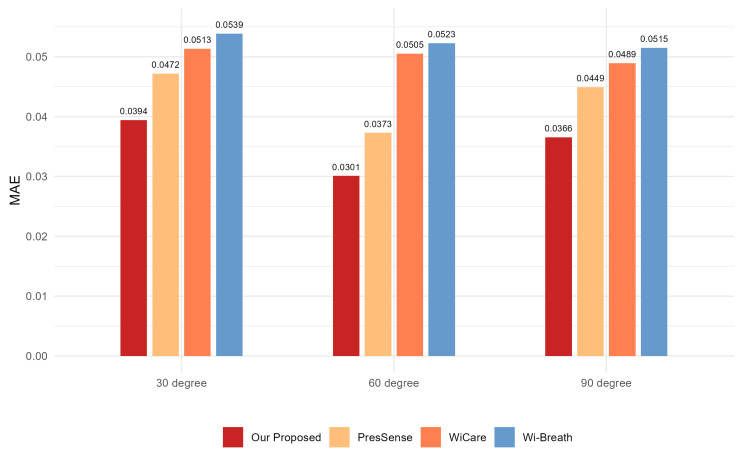
Comparison of MAE for four breathing monitoring methods in three different orientations.

**Figure 15:**
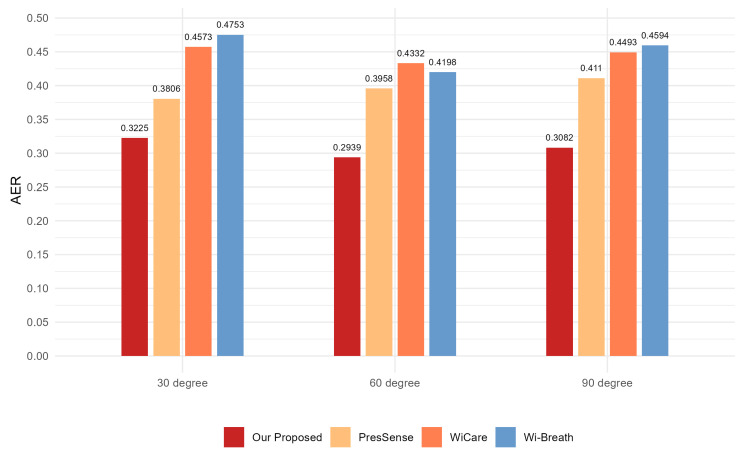
Comparison of AER for four breathing monitoring methods in three different orientations.

In the experimental setup for this section, the three transmitting antennas and the receiving antenna were located in a straight line, and the antennas were spaced 0.5 m apart from each other. The transmitting and receiving ends were 3 m apart. The vertical distance between the human chest position and the center of the $Tx_{1}$ and $Rx_{1}$ connecting lines was 1 m. The position of the human chest remained the same; only the orientation changed, by 90 degrees, 60 degrees, and 30 degrees from the vertical, respectively. A schematic of the three experimental scenarios can be seen in Figure 12:

Figure 13, Figure 14 and Figure 15 show the ACC, MAE, and AER results of the proposed method under three different orientation conditions, respectively. By comparing and analyzing these graphs, it is clear that our method achieved the highest accuracy and low MAE values for breathing detection. This result indicates a high degree of consistency between the breathing signals extracted using our WiFi device-based method and the breathing signals detected by the wearable device sensors. Meanwhile, the AER of our method was kept below 0.35 bpm in all test orientations, which further validated the validity and reliability of this method for breathing monitoring. In addition, the monitoring results showed that the most significant effect was observed when the observation was performed at an angle of 60 degrees. This phenomenon can be attributed to the fact that the projected area of the chest cavity in the reflected signal increased at this angle, which improved the monitoring efficiency of the breathing signal. On the contrary, when observing at an angle of 30 degrees, part of the reflected signals from the chest may have been obstructed by other parts of the body, such as the arms, resulting in the interference of these signals during re-reflection, which, in turn, reduced the accuracy of the monitoring.

#### 4.3.3. Assessment of Different Positions of the Human Chest

**Figure 16:**
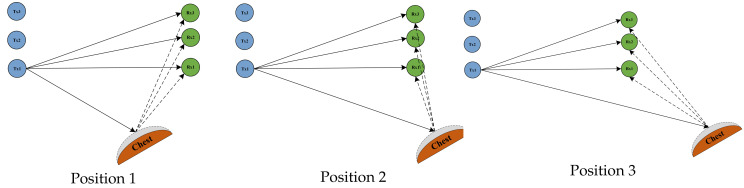
Schematic illustration of a scenario in which the human chest is in position 1, position 2, and position 3.

**Figure 17:**
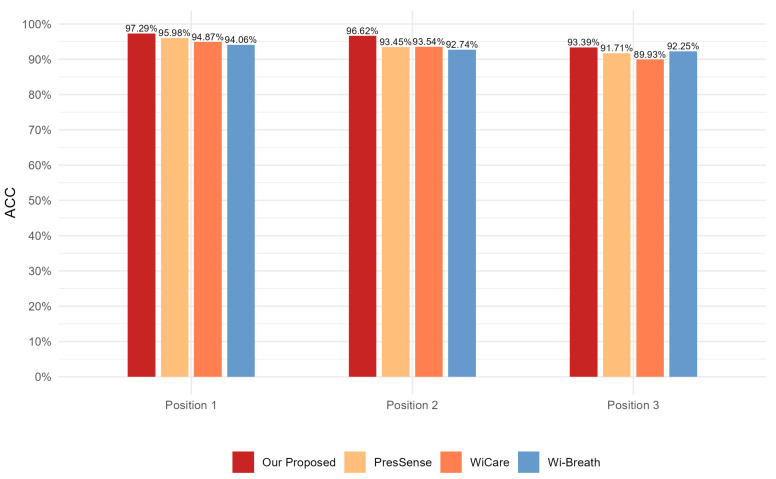
Comparison of ACC for four breathing monitoring methods in three different positions.

**Figure 18:**
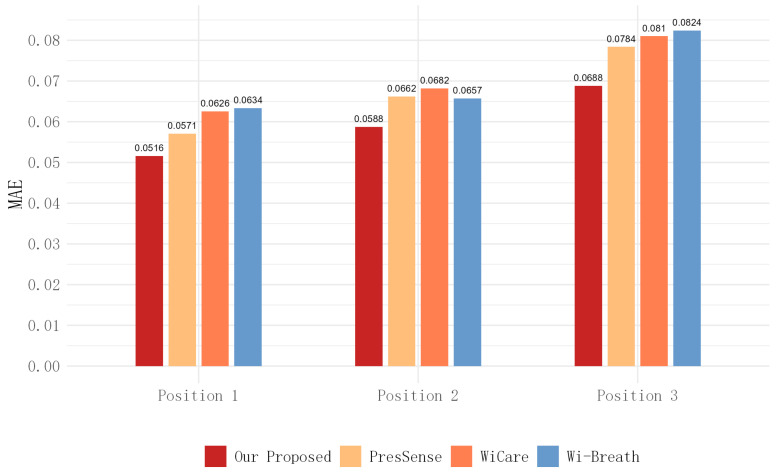
Comparison of MAE for four breathing monitoring methods in three different positions.

**Figure 19:**
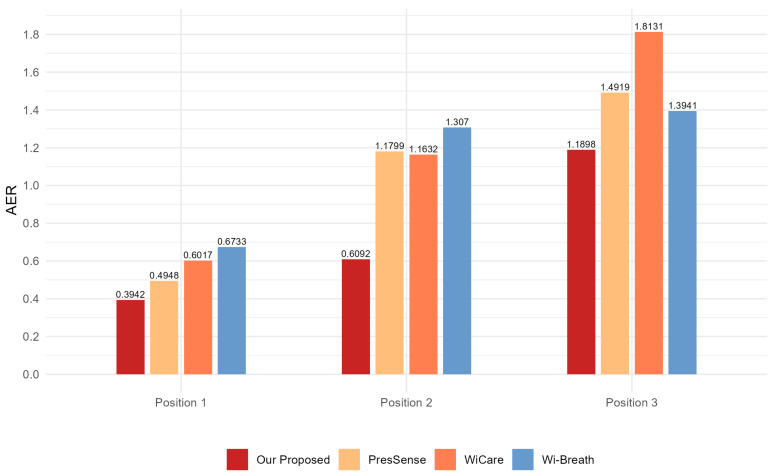
Comparison of AER for four breathing monitoring methods in three different positions.

In the experimental setup for this section, the human chest orientation was kept constant at an angle of 60 degrees from vertical. The human chest was positioned at a vertical distance of 1 m from the center of the $Tx_{1}$ and $Rx_{1}$ lines. The position of the human chest was changed to 1 m, 2 m, and 3 m horizontally from the center of the $Tx_{1}$ and $Rx_{1}$ connection lines, respectively. The three experimental scenarios are illustrated in Figure 16:

Figure 17, Figure 18 and Figure 19 show the ACC, MAE, and AER results obtained from the respiration monitoring method proposed in this study at different monitoring locations. By carefully comparing these graphs, it can be found that even at different monitoring locations, although the errors of the breathing signals extracted using the proposed method varied, the proposed method still achieved a lower MAE compared with the existing techniques. In addition, as the monitoring distance increased, although the accuracy of all four monitoring methods showed a decreasing trend and the AER value increased, the proposed method maintained accuracy above 93% and an error rate below 1.2 bpm in most cases. This result indicates that a WiFi breathing monitoring system based on the method of this study can operate effectively with acceptable accuracy even at greater distances. This effectively confirms the high accuracy and reliability of the proposed method for breathing monitoring.

## 5. Conclusions

In this paper, we propose a novel method aimed at improving the accuracy of breathing monitoring based on WiFi signals. Our approach involves tracking the trajectory of CSI ratio samples on the I/Q plane, to mitigate the effects of channel variability and complexity, thereby achieving high-precision breathing monitoring. The method in this study ensures the temporal continuity and accuracy of the CSI data by eliminating static object reflection signals in the preprocessing stage, applying a Gaussian regression process to interpolate the time series, and using a CSI ratio model to remove the random phase offsets. We developed an algorithm based on discretization analysis for detecting CSI ratio sample points on a complex plane representing large human movements, thus excluding the interference of large movements in extracting respiratory signals. Ultimately, the variation of the CSI ratio is smoothed by the spline curve fitting technique, and a breathing signal extraction algorithm is proposed, to realize breathing detection. Our experimental results demonstrated that our proposed method outperforms the existing approaches, in terms of accuracy and reliability. In the current era of the Internet of Things, our method holds significant importance for remote health monitoring in domains such as smart homes, smart offices, and smart healthcare.

It is worth noting that while our proposed method effectively and accurately monitors human breathing, its performance is optimal only when the subject remains still and experiences minimal movement during the breathing process. Continuous subject activity significantly reduces the monitoring accuracy of all methods, including ours. To address this limitation, future research will explore novel approaches, such as introducing ray tracing models or utilizing fusion technology with multiple antennas from different angles.

## Figures and Tables

**Table 1:** CSI collection system hardware parameters.

Parameter Category	Parameter Details
Brand and model	TP-Link TL-WDR4310 v1.0
SoC	Atheros AR9344
CPU frequency (MHz)	560
Flash size (MB)	8
RAM size (MB)	128
WLAN hardware	Atheros AR9344,Atheros AR9580
Supported WLAN modes (2.4 GHz)	b/g/n
Supported WLAN modes (5.0 GHz)	a/n
Processor architecture	MIPS 74Kc
Wireless interface 1	SoC integrated NIC Atheros AR9340 2.4 GHz
Wireless interface 2	Independent NIC Atheros AR9580 5 GHz

## Data Availability

The data presented in this study are available on request from the corresponding author. The data are not publicly available, due to privacy.
